# Supplementary material for: Description and phylogenetic relationships of a new species of Torvoneustes (Crocodylomorpha, Thalattosuchia) from the Kimmeridgian of Switzerland
Source: PeerJ. 2023 Jul 19;11:e15512. doi: 10.7717/peerj.15512 (PMC10362849; doi:10.7717/peerj.15512)
Supplement: Supplementary file 1 [file peerj-11-15512-s002.odt]

| Model IDs | Description |  |
| --- | --- | --- |
| M3#1037 | Left dentary (3 meshes) | http://morphomuseum.com/Specimenfiles/sendFile/1037/bf5c05 |
| M3#1038 | Right dentary (3 meshes) | http://morphomuseum.com/Specimenfiles/sendFile/1038/0f9aa0 |
| M3#1039 | Left ramus (2 meshes) | http://morphomuseum.com/Specimenfiles/sendFile/1039/b453bd |
| M3#1040 | Right ramus (3 meshes) | http://morphomuseum.com/Specimenfiles/sendFile/1040/51b0be |
| M3#1041 | Left splenial (2 meshes) | http://morphomuseum.com/Specimenfiles/sendFile/1041/b88b9d |
| M3#1042 | Right splenial (2 meshes) | http://morphomuseum.com/Specimenfiles/sendFile/1042/8e1d9f |
| M3#1043 | Frontal and left prefrontal | http://morphomuseum.com/Specimenfiles/sendFile/1043/cdc6a5 |
| M3#1044 | Left maxilla (4 meshes) | http://morphomuseum.com/Specimenfiles/sendFile/1044/6734ec |
| M3#1045 | Right maxilla | http://morphomuseum.com/Specimenfiles/sendFile/1045/5e4938 |
| M3#1046 | Left nasal | http://morphomuseum.com/Specimenfiles/sendFile/1046/8461f4 |
| M3#1047 | Right nasal | http://morphomuseum.com/Specimenfiles/sendFile/1047/258597 |
| M3#1048 | Parietal | http://morphomuseum.com/Specimenfiles/sendFile/1048/140093 |
| M3#1049 | Right postorbital | http://morphomuseum.com/Specimenfiles/sendFile/1049/ced2e2 |
| M3#1050 | Right prefrontal | http://morphomuseum.com/Specimenfiles/sendFile/1050/9b4a86 |
| M3#1051 | Right premaxilla | http://morphomuseum.com/Specimenfiles/sendFile/1051/d903cc |
| M3#1052 | Left squamosal | http://morphomuseum.com/Specimenfiles/sendFile/1052/e78979 |
| M3#1053 | Right squamosal | http://morphomuseum.com/Specimenfiles/sendFile/1053/6560c4 |
| M3#1054 | Reconstruction of the mandible | http://morphomuseum.com/Specimenfiles/sendFile/1054/3c4fb0 |
| M3#1055 | Reconstruction of the cranium | http://morphomuseum.com/Specimenfiles/sendFile/1055/e68720 |
